# Connectivity and Functionality of the Globus Pallidus Externa Under Normal Conditions and Parkinson's Disease

**DOI:** 10.3389/fncir.2021.645287

**Published:** 2021-03-02

**Authors:** Jie Dong, Sarah Hawes, Junbing Wu, Weidong Le, Huaibin Cai

**Affiliations:** ^1^Laboratory of Neurogenetics, Transgenic Section, National Institute on Aging, National Institutes of Health, Bethesda, MD, United States; ^2^Child Health Institute of New Jersey, Rutgers University, New Brunswick, NJ, United States; ^3^Liaoning Provincial Center for Clinical Research on Neurological Diseases & Liaoning Provincial Key Laboratory for Research on the Pathogenic Mechanisms of Neurological Diseases, The First Affiliated Hospital of Dalian Medical University, Dalian, China; ^4^Medical School of University of Electronic Science and Technology of China, Institute of Neurology, Sichuan Provincial Hospital, Sichuan Academy of Medical Science, Chengdu, China

**Keywords:** globus pallidus externa, Parkinson's disease, basal ganglia, prototypic neurons, arkypallidal neurons, dopaminergic neurons, glia

## Abstract

The globus pallidus externa (GPe) functions as a central hub in the basal ganglia for processing motor and non-motor information through the creation of complex connections with the other basal ganglia nuclei and brain regions. Recently, with the adoption of sophisticated genetic tools, substantial advances have been made in understanding the distinct molecular, anatomical, electrophysiological, and functional properties of GPe neurons and non-neuronal cells. Impairments in dopamine transmission in the basal ganglia contribute to Parkinson's disease (PD), the most common movement disorder that severely affects the patients' life quality. Altered GPe neuron activity and synaptic connections have also been found in both PD patients and pre-clinical models. In this review, we will summarize the main findings on the composition, connectivity and functionality of different GPe cell populations and the potential GPe-related mechanisms of PD symptoms to better understand the cell type and circuit-specific roles of GPe in both normal and PD conditions.

## Background

**Figure 1:**
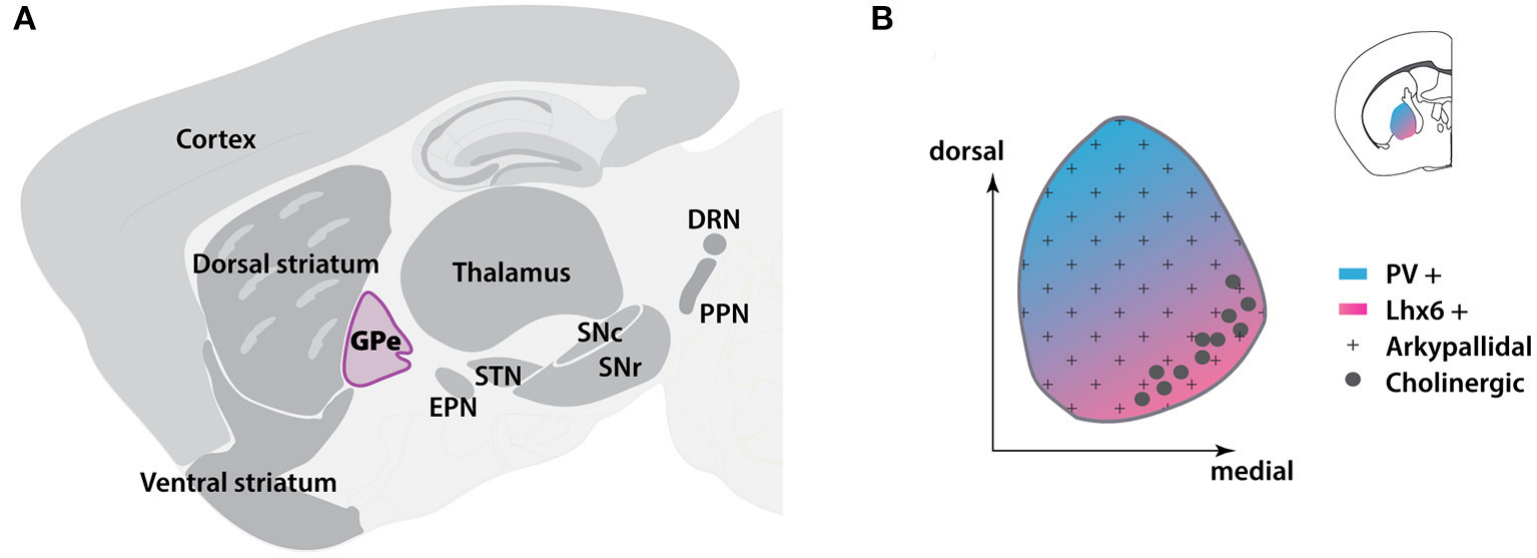
Location and cellular distribution of GPe in mouse brain. **(A)** Sagittal scheme showing the locations of BG nuclei (dark gray) and other subregions in the brain. The GPe is located in the central BG. **(B)** Location of GPe (inset) and spatial distribution of GPe subpopulations at the coronal level. BG, basal ganglia; DRN, dorsal raphe nucleus; EPN, entopeduncular nucleus; GPe, globus pallidus externa; Lhx6, LIM homeobox 6; PPN, pedunculopontine nucleus; PV, Parvalbumin; SNc, substantia nigra pars compacta; SNr, substantia nigra pars reticulata; STN, subthalamic nucleus. The template of brain atlas was obtained from Allen Mouse Brain Atlas.

The globus pallidus externa (GPe) resides in the center of the basal ganglia (BG) (Figure 1A) and is traditionally considered a relay station of the indirect pathway in the BG circuits (Albin et al., 1989; Calabresi et al., 2014). According to the classical model, the GPe receives the majority of inputs from the striatum, and subsequently transfers the information to the subthalamic nucleus (STN) and the BG output nuclei, which include the substantia nigra pars reticulata (SNr), globus pallidus interna [GPi, the equivalent structure to rodent entopeduncular nucleus (EPN)] (Gerfen et al., 1990; Smith et al., 1998; Kita, 2007). However, accumulating studies have demonstrated that the GPe can coordinate and respond to more brain regions than the indirect pathway itself. Direct connections have been found between the GPe and cortex, thalamus or pedunculopontine nucleus (Lavoie and Parent, 1994; Sato et al., 2000; Yasukawa et al., 2004; Mallet et al., 2012; Milardi et al., 2015; Saunders et al., 2015; Eid et al., 2016). With discovery of the heterogeneity of different neuron subtypes, GPe is no longer considered as a simple homogenous nucleus in the brain. The GPe neurons can be divided into at least two subpopulations: the prototypic and arkypallidal GPe neurons (Figure 1B) (Mallet et al., 2012; Abdi et al., 2015; Dodson et al., 2015). Furthermore, even within each subpopulation, several distinct neuronal subtypes exist that are characterized by distinct expression patterns of molecular markers (Bevan et al., 1998; Cooper and Stanford, 2000; Mastro et al., 2014; Abdi et al., 2015; Hernández et al., 2015). Each subtype has its own unique anatomical projections, electrophysiological properties, and functions (Mastro et al., 2014, 2017; Abdi et al., 2015; Hernández et al., 2015; Glajch et al., 2016; Pamukcu et al., 2020). This suggests that the GPe is a dynamic and complex information hub, rather than a simple relay station in the BG. Functionally, GPe plays a critical role in regulating motor and non-motor activities. Abnormal GPe neuron activity has been observed in Parkinson's disease (PD) and other movement disorders, such as Huntington's disease and dystonia (Albin et al., 1992; Hutchison et al., 1994; Bevan et al., 2002; Merello et al., 2004; Chan et al., 2011; Ligot et al., 2011). In this review, we will highlight the central processing role of GPe in BG and provide an update on the connectivity and functionality of GPe neuron subpopulations, especially in the PD condition. We will also discuss the GPe's functions in motor and non-motor regulation and its potential value in clinical diagnosis and treatment of PD.

## Main Text

### GPe Serves as a Central Hub in the BG Network

BG participates in a variety of functional processes, including motor generation, motor control, motor learning, and value-based decision making (Graybiel et al., 1994; Doyon et al., 2009; Ito and Doya, 2009). Generally, the structures of the BG include the input nuclei striatum and STN, relay stations GPe and STN, and output nuclei GPi and substantia nigra (SN). Distinct striatal GABAergic spiny projection neurons (SPNs) give rise to two classical pathways, that convey cortical instructions either directly or indirectly to the BG outputs (Gerfen et al., 1990; Gerfen and Surmeier, 2011; Calabresi et al., 2014). The direct pathway SPNs (dSPNs) project directly to the output nuclei SNr and GPi. The indirect pathway SPNs (iSPNs) send axons to the intermediate nuclei GPe, which then relay striatal inputs to the output nuclei. It is generally accepted that the dSPNs express D1 dopamine receptors (DRD1) and the iSPNs express D2 dopamine receptors (DRD2). The DRD1 and DRD2 are associated with distinct G protein-coupled intracellular signaling transduction pathways (Gerfen et al., 1990; Gerfen and Surmeier, 2011). In the indirect pathway, the GPe receives the inhibitory GABAergic inputs from iSPNs, which suppresses the activity of GABAergic GPe neurons and leads to a disinhibition of the GPe-targeted STN glutamatergic neurons. The excitatory STN neurons subsequently activate the SNr GABAergic outputs to inhibit the activity of thalamus and brainstem, resulting in suppression of motor activity. On the contrary, in the direct pathway, the GABAergic SNr neurons are directly inhibited by the GABAergic dSPNs inputs, which leads to a disinhibition of thalamic and brainstem activity and subsequent increase of motor activity.

While the typical BG functions have been classified as motor-facilitating or motor-inhibiting based on the activation of the direct or indirect pathway (Albin et al., 1989), recent studies challenge this model and suggest that the direct and indirect pathways are concomitantly activated in motor execution (Cui et al., 2013; Jin et al., 2014; Yttri and Dudman, 2016; Meng et al., 2018). At the circuit level, the striatal projections to the GPe do not strictly originate from the iSPNs. Single-cell tracing studies in rats found that a majority of dSPNs extend their axon collaterals to the GPe (Kawaguchi et al., 1990; Wu et al., 2000). Those dSPN axon collaterals may function as a bridge to mediate the crosstalk between the direct and indirect pathways in the GPe (Cazorla et al., 2014). Besides the local microcircuit modulation of the dSPNs and iSPNs in the striatum, the bridge function of GPe may also partly contribute to the concomitant activation of the classical pathways, acting as a concurrent “brake” when the “accelerator” direct pathway is activated (Graybiel, 2000; Cazorla et al., 2014). Therefore, the BG circuits are more complicated than what the basic model describes.

In addition to the direct and indirect pathways, multiple networks also participate in regulating BG function, such as the hyper-direct (Cortex-STN) pathway, pallidostriatal pathway, GPe-STN loop, and GPe-Cortex loop (Bevan et al., 2002; Nambu et al., 2002; Mallet et al., 2016; Abecassis et al., 2020). GPe exerts critical roles in all of these pathways, which provides the foundation for its central position in guiding the information flow through the BG networks. These pathways will be discussed in further detail in the following sections.

### Distinct Cell Types in GPe

**Figure 2:**
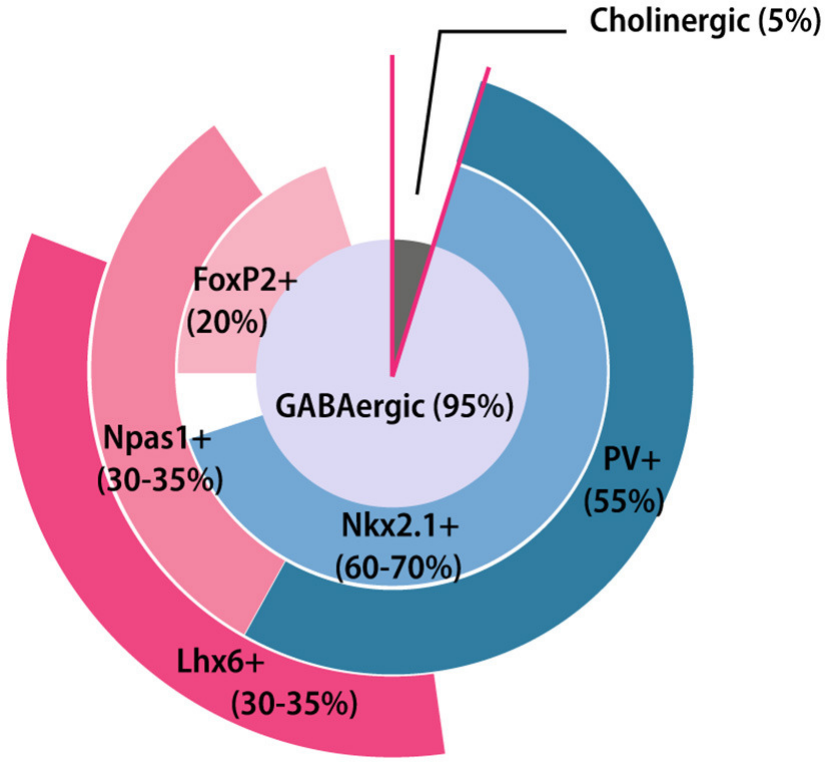
GPe neuron composition. Pie chart summarizing the GPe neuronal composition and general overlapping relationships among different molecular markers. The area of each marker represents the approximate percentage of each subpopulation. FoxP2+, Forkhead box protein P2-positive; Lhx6+, LIM homeobox 6-positive; Nkx2.1+, Nk2 homeobox 1-positive; Npas1+, Neuronal PAS domain protein 1-positive.

GPe contains predominantly GABAergic neurons (about 95%) and a small number of cholinergic neurons (Hegeman et al., 2016) (Figure 2). Interestingly, a few recent studies have indicated that the GPe cholinergic neurons also release GABA (Tkatch et al., 1998; Saunders et al., 2015), which blurs the boundary between the roles of GABAergic and cholinergic neurons. Because cholinergic neurons possess distinct morphological and electrophysiological properties, we will discuss them separately. Additionally, as glial cells are also enriched in the GPe, we will also discuss the distribution and function of glia in this section.

#### Molecular Architecture of GABAergic GPe Neurons

GABAergic neurons in the GPe can be divided into two subtypes: prototypic and arkypallidal neurons. The prototypical neurons occupy the largest portion of GPe, project mostly to the STN, and exhibit fast and regular firing rates *in vivo*. The arkypallidal neurons project primarily to the striatum, and present slower and irregular firing rates (Mallet et al., 2012; Abdi et al., 2015; Dodson et al., 2015). The cellular heterogeneity of GPe neurons start from embryonic stages (Nóbrega-Pereira et al., 2010). Generally, prototypic neurons are primarily derived from the medial ganglionic eminence and arkypallidal neurons arise from the lateral ganglionic eminence or caudal ganglionic eminence (Nóbrega-Pereira et al., 2010; Dodson et al., 2015; Abecassis et al., 2020). However, to date, we still lack a comprehensive molecular description for both prototypic and arkypallidal neurons.

The prototypic neurons have diverse and partially overlapping molecular expression profiles (Nóbrega-Pereira et al., 2010; Mastro et al., 2014; Abdi et al., 2015; Dodson et al., 2015; Abrahao and Lovinger, 2018; Abecassis et al., 2020). Nkx2.1 (Nk2 homeobox 1) is a useful marker for the prototypic neurons, accounting for ~60–70% of the total GPe neuron population (Dodson et al., 2015; Abecassis et al., 2020). Among them, at least two-thirds of Nkx2.1-positive (Nkx2.1^+^) neurons co-express the calcium-binding protein parvalbumin (PV) (Abdi et al., 2015; Dodson et al., 2015). Conversely, almost all PV-positive (PV^+^) neurons express Nkx2.1 (Abdi et al., 2015). PV^+^ neurons (about 55% of all the GPe neurons) are mainly concentrated at the dorsolateral part of GPe (Abrahao and Lovinger, 2018) (Figure 1B). The principle electrophysiological signature of PV^+^ neurons are their high firing rates with small sag ratio and membrane resistance, making them the most physiologically distinct subpopulation (Mastro et al., 2014; Hernández et al., 2015; Abrahao and Lovinger, 2018; Abecassis et al., 2020). Another subset of Nkx2.1^+^ neurons express LIM homeobox 6 (Lhx6). The Lhx6-positive (Lhx6^+^) neurons account for approximately one-third of GPe neurons and most of them are Nkx2.1^+^ (Mastro et al., 2014; Abrahao and Lovinger, 2018; Abecassis et al., 2020). Lhx6^+^ neurons are more concentrated in the medial and ventral parts of GPe, showing a relatively reverse distribution pattern compared to the PV^+^ neurons (Abrahao and Lovinger, 2018; Abecassis et al., 2020) (Figure 1B). However, some PV^+^ neurons also display weak Lhx6 immunoreactivity. The reported overlap of Lhx6^+^ and PV^+^ neurons varies widely across laboratories and studies (Mastro et al., 2014, 2017; Dodson et al., 2015; Hernández et al., 2015; Abrahao and Lovinger, 2018; Abecassis et al., 2020). Several studies have found that about 25% of Lhx6^+^ neurons are also PV^+^ (Hernández et al., 2015; Abrahao and Lovinger, 2018; Abecassis et al., 2020), while others show the overlapping percentage is up to 50% (Dodson et al., 2015; Mastro et al., 2017). In addition, about 25–35% Lhx6^+^ neurons also co-express neuronal PAS domain protein 1 (Npas1) (Abrahao and Lovinger, 2018; Abecassis et al., 2020), but not all Npas1^+^ neurons are Lhx6^+^. The intrinsic electrophysiological properties within Lhx6^+^ neurons are varied and predictably based on the intensity of Lhx6 immunoreactivity. Weak Lhx6-expressing neurons are more inclined to present similar electrophysiological characteristics as PV^+^ neurons, while neurons strongly expressing Lhx6 exhibit lower firing rates and higher sag ratio (Abecassis et al., 2020). Although both PV and Lhx6 are biomarkers for prototypical neurons, the connectivity and functions of these neurons are different (Mastro et al., 2014, 2017).

Compared to prototypic neurons, arkypallidal neurons have a clearer molecular architecture manifested with the distinctive expression of opioid precursor preproenkephalin and forkhead box protein P2 (FoxP2) (Mallet et al., 2012; Abdi et al., 2015; Abrahao and Lovinger, 2018). The arkypallidal neurons are distributed evenly across the GPe subregions and present electrophysiological characteristics of lower firing rates and larger sag and input resistance (Abdi et al., 2015; Hernández et al., 2015; Glajch et al., 2016). FoxP2 is a highly selective marker for the arkypallidal, with no overlap occurring with the prototypic marker, Nkx2.1 (Abdi et al., 2015; Abecassis et al., 2020). Embryonically, FoxP2^+^ neurons are derived largely from the lateral ganglionic eminence and modestly from the caudal ganglionic eminence and medial ganglionic eminence (Ferland et al., 2003; Long et al., 2009; Dodson et al., 2015). Most FoxP2^+^ neurons co-express Npas1 (Abdi et al., 2015; Dodson et al., 2015; Abecassis et al., 2020) at ratios ranging from 80% (Abecassis et al., 2020) to 100% (Abdi et al., 2015). Npas1^+^ neurons account for one third of GPe neurons, and do not overlap with PV^+^ expression (Hernández et al., 2015; Abecassis et al., 2020). Npas1^+^ neurons can be divided into two subgroups based on Nkx2.1 expression (Flandin et al., 2010; Dodson et al., 2015; Abrahao and Lovinger, 2018; Abecassis et al., 2020). A recent tracing experiment revealed that the Nkx2.1^+^/Npas1^+^ neurons are a distinct GPe subclass which project to the cortex (Abecassis et al., 2020).

#### Cholinergic GPe Neurons

The choline acetyltransferase (ChAT)-expressing cholinergic neurons account for only a small proportion of GPe. These neurons are concentrated in the medial and ventral border of GPe and exhibit distinctly large soma and axon arbor size, making them easily recognizable (Saunders et al., 2015; Guo et al., 2016). Historically, the GPe cholinergic neurons are considered to be an extension of the nucleus basalis caudal to the pallidum (Mckinney et al., 1983; Mesulam et al., 1984). However, recent studies have demonstrated that these cholinergic neurons exhibit connectivity similar to other GPe neurons, which project heavily to the frontal cortex and receive innervations from the dorsal striatum and STN (Grove et al., 1986; Hernández et al., 2015; Saunders et al., 2015; Ährlund-Richter et al., 2019). In addition, one recent study reported that half of the cholinergic neurons also express Lhx6 (Abecassis et al., 2020), which further supports the notion that GPe cholinergic neurons are distinct from the those in nucleus basalis. In terms of their electrophysiological properties, the cholinergic neurons become spontaneously active around sexual maturity. Once active, they exhibit longer-duration action potentials and very low firing rates (McKenna et al., 2013; Hernández et al., 2015; Saunders et al., 2015).

#### Glia Are an Integral Part of GPe

Neurons are not the only cell type in the GPe, and in fact, glia are the most numerous GPe cells (Lange et al., 1976). The astrocyte-to-neuron ratio in the GPe is roughly two-to-one (Cui et al., 2016). Furthermore, the astrocytes have a higher density within the GPe compared to neighboring brain subregions (Charron et al., 2014). GPe astrocytes express a myriad of receptors and transports, such as DRD2, glutamate transporters and receptors, and GABA transporters (GATs), of which GAT-3 is expressed exclusively in the processes of GPe astrocytes (Galvan et al., 2005, 2010; Nedergaard and Verkhratsky, 2012; Araque et al., 2014). Ultrastructural studies show that GPe astrocyte processes are juxtaposed to striatopallidal terminals (Galvan et al., 2010). Recent studies suggest that GPe astrocytes participate in the striatopallidal pathway through local modification of striatal GABAergic synapses and through regulating the expression and activity of metabotropic glutamate receptors (mGluRs) and GAT-3 (Cui et al., 2016; Chazalon et al., 2018). Generally, there remains much space for researchers to explore the biological function of GPe glia cells and their relevance in disease.

### Afferent Projections to the GPe

#### Striatum

**Figure 3:**
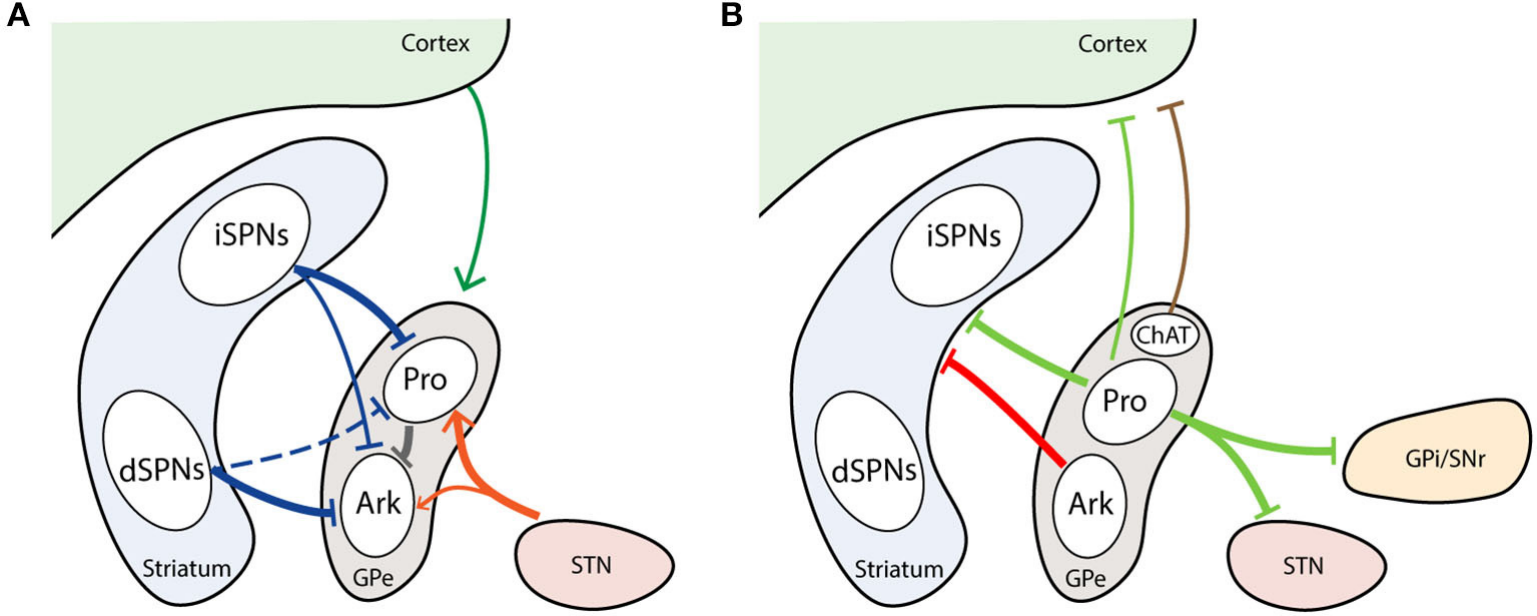
Major inputs and outputs of GPe neuron subtypes. **(A)** GPe afferents from striatum, cortex, and STN. **(B)** GPe efferents to striatum, cortex and BG downstream nuclei. Thickness of line represent the intensity of projections. Dash line marks unconfirmed connections. Arrowhead represents excitatory projection. T-head depicts inhibitory projection. Ark, arkypallidal neurons; ChAT, choline acetyltransferase; Pro, prototypic neurons.

In the classical model of the BG circuit, the GPe receives inputs preferentially from striatum. The GPe possesses motor, associative and limbic subdivisions, which link to the corresponding territories in the cortex and striatum (Saga et al., 2017). Both striatum and GPe are topographically organized structures, as are the projections between them (Heilbronner et al., 2018). In primates, 90% of striatopallidal projections follow the dimensional topography, that is lateral to lateral, dorsal to dorsal, and caudal to caudal striatum to GPe, respectively (Hedreen and Delong, 1991; Heilbronner et al., 2018). Because the striatum contains many more neurons than the GPe, one GPe neuron may receive inputs from tens to hundreds of SPNs, resulting in tens of thousands of striatopallidal boutons (small swellings at axon terminals) (Oorschot, 1996). As mentioned previously, the GPe receives GABAergic inputs from both iSPNs and dSPNs (Figure 3A). On average, the number of boutons formed by an axon of an iSPN is twice that of an dSPN in rats (Kawaguchi et al., 1990). In terms of the inputs from striosome and matrix compartments, the axon of matrix iSPNs often makes two or more bushy arborizations in GPe, while striosome iSPN axons do not make multiple arborizations. On the other hand, the axon collaterals from both the matrix and striosome dSPNs rarely make multiple bushy arborizations in GPe (Fujiyama et al., 2011). The inhibitory striatal inputs to GPe also differ between prototypic and arkypallidal neurons. Computational modeling has predicted that inputs to prototypic neurons are much stronger than to arkypallidal neurons (Nevado-Holgado et al., 2014). Several studies have suggested that iSPNs strongly inhibit prototypic neurons (Yuan et al., 2017; Aristieta et al., 2020; Silberberg and Ketzef, 2020). A recent study further demonstrates that arkypallidal neurons receive weaker and less projections from iSPNs compared to prototypic neurons (Aristieta et al., 2020). However, there still exists uncertainty regarding the GPe subpopulations that dSPNs innervate. One study found that dSPNs selectively inhibit arkypallidal neurons (Silberberg and Ketzef, 2020), while another study demonstrates that substance P, primarily expressed in dSPNs, exclusively affects prototypic neurons (Mizutani et al., 2017). In addition to the GABAergic neurons, striatopallidal inputs also target cholinergic neurons in GPe, but the specific function of this innervation has not yet been determined (Saunders et al., 2015).

#### STN

The principle glutaminergic inputs to the GPe are from the STN (Smith and Parent, 1988). The STN is also a topographic structure with three subdivisions, corresponding to motor, limbic, and associative functions. Neurons projecting to the GPe are present in the dorsolateral part of the STN, which is defined as the motor subdivision (Alkemade et al., 2015). In the rat brain, each STN neuron can form more than 400 boutons in GPe (Koshimizu et al., 2013). Taking into consideration the abundance of GPe and STN neurons, each GPe neuron may receive on average 135 boutons from STN neurons (Koshimizu et al., 2013; Kita and Jaeger, 2016), which is much fewer than the striatal inputs. Computational modeling indicated that the STN targets a subset of Npas1^+^ neurons, but this has yet to be confirmed in animal models (Bogacz et al., 2016). However, two recent studies have demonstrated that the STN gives rise to stronger inputs to PV^+^ neurons than Npas1^+^ neurons (Pamukcu et al., 2020; Silberberg and Ketzef, 2020). Aristieta et al. also found that STN preferentially activates prototypic neurons (Aristieta et al., 2020) (Figure 3A). The typical excitatory response in GPe following STN stimulation is mediated by ionotropic α-amino-3-hydroxy-5-methylisoxazole-4-propionic acid (AMPA), N-methyl-D aspartic acid (NMDA) receptors and kainate receptors (Eid and Parent, 2016). In addition, the mGluRs are also involved in excitatory postsynaptic responses in GPe (Stefani et al., 1998; Conn et al., 2005; Cui et al., 2016).

#### Cortex

Since the discovery of cortical projection to GPe in 1977, an increasing number of studies have started to focus on the direct corticopallidal pathway (Leichnetz and Astruc, 1977; Naito and Kita, 1994; Hunt et al., 2018; Karube et al., 2019; Magno et al., 2019; Abecassis et al., 2020). The corticopallidal axonal collaterals are also topographically organized, but are sparser than the corticostriatal projections (Naito and Kita, 1994; Karube et al., 2019). The frontal and motor projections target more rostral areas, whereas the sensory projections target more posterior areas of the GPe (Hintiryan et al., 2016; Hooks et al., 2018). This corticopallidal pathway has been confirmed in multiple species, including rat, lamprey, monkey, and human (Leichnetz and Astruc, 1977; Naito and Kita, 1994; Stephenson-Jones et al., 2013; Grewal et al., 2018; Karube et al., 2019). In rats, the density of cortical boutons in the GPe is nearly 50% that of cortical bouton density in the striatum (Karube et al., 2019). These boutons preferentially innervate FoxP2^+^ arkypallidal neurons (Karube et al., 2019). In contrast, only one-third to half of the more heterogeneous prototypic neurons receive cortical inputs (Karube et al., 2019) (Figure 3A). Motor cortex is the main origin of corticopallidal axons. M1 terminals are most often located within the dorsal GPe, while M2 terminals are more likely to cluster in the ventral GPe (Karube et al., 2019). Some studies suggest that the GPe-projecting cortical neurons are predominantly the pyramidal tract type, which locates in the lower layer 5 in the cortex and usually produce collaterals to the striatum (Economo et al., 2016; Abecassis et al., 2020).

#### Thalamic Intralaminar Nuclei

Thalamic intralaminar nuclei consist of the central median and parafascicular (Pf) nuclei. The thalamus projects topographically to the GPe in parallel with the thalamostriatal projections (Kincaid et al., 1991). One anterograde tracing experiment indicated that the lateral part of Pf innervates the middle territory of GPe, while the medial part of Pf innervates the caudomedial territory of GPe (Yasukawa et al., 2004). Recent computational analysis suggests that Npas1^+^ neurons in GPe are the major target of thalamic inputs (Nevado-Holgado et al., 2014). Much work remains to be done to fully appreciate the connections of thalamopallidal projections.

#### Brainstem Nuclei

In addition to inputs from the forebrain, GPe also receives dopaminergic inputs directly from the midbrain, cholinergic inputs from the pedunculopontine tegmental nucleus and serotonergic inputs from the dorsal raphe nucleus (DRN) (Eid and Parent, 2016). Several studies have shown that dopamine (DA) inputs arise from subpopulations scattered within the substantia nigra pars compacta (SNc), and to a lesser extent within the ventral tegmental (VTA) and retrorubral areas (Charara, 1994; Jan et al., 2000; Prensa and Parent, 2001; Dopeso-Reyes et al., 2014). Indeed, VTA projects predominately to the ventral pallidum, as a part of processing motivation (Melendez et al., 2004). DA innervation is not uniform throughout the GPe, of which the anterior and dorsal parts of GPe exhibit a higher density of DA axon varicosities (Eid and Parent, 2015, 2016). These afferents are considered to be collaterals from the nigrostriatal pathway, with only a few from distinct nigropallidal projections (Smith and Villalba, 2008; Bouali-Benazzouz et al., 2009; Rommelfanger and Wichmann, 2010). D2-class DA receptors (DRD2, DRD3, DRD4) are prominently expressed in the GPe (Richfield et al., 1987; Smith and Villalba, 2008; Mamad et al., 2015). DRD2 mRNA is present in all GPe neurons, but striatum-projecting GPe neurons express higher DRD2 levels than the neurons which project to the STN (Hoover and Marshall, 2004). DRN axons project to almost all the BG nuclei (Vertes, 1991) and GPe is no exception. The GPe receives serotonergic projections mostly from the ventrolateral part of the DRN, which mainly takes part in the behavioral reactions to stress stimuli (Gorbachevskaya and Saulskaya, 2019). In turn, DRN projects more heavily to the associative territory than the limbic or sensorimotor territories in GPe (Eid et al., 2013). So far, at least five subtypes of 5-hydroxytryptamine (5-HT) serotonin receptors are expressed in GPe, including 5-HT_1A_, 5-HT_1B_, 5-HT_2A_, 5-HT_4_, and 5-HT_7_, located at either postsynaptic or presynaptic sites (Varnäs et al., 2004; Kita, 2007; Riahi et al., 2013; Miguelez et al., 2014; Eid and Parent, 2016). The postsynaptic 5-HT_4_ or 5- HT_7_ receptors mediate stimulatory effects in GPe neurons (Chen et al., 2008; Hashimoto and Kita, 2008), whereas the presynaptic 5-HT_1B_ receptors reduce the release of glutamate and GABA from subthalamopallidal and striatopallidal terminals, respectively (Castro et al., 1998; Querejeta et al., 2005).

### Efferent Projections of GPe

#### BG Downstream Nuclei

It is generally considered that GPe prototypic neurons project to the downstream BG nuclei (Mallet et al., 2012; Abdi et al., 2015). On average, a single GPe neuron forms at least 6 boutons on the soma and proximal dendrites of STN neurons (Baufreton et al., 2009). PV^+^ neurons occupy more than 90% of the pallidal efferent to STN, and Lhx6^+^ neurons account for about 40% (Mastro et al., 2014; Hernández et al., 2015). The innervation density and distribution are different between PV^+^ and Lhx6^+^ neurons. The PV^+^ projections display different topographic properties, in which the rostroventral PV^+^ neurons project to the central portion of the STN, while the caudal PV^+^ neurons project to the periphery of the STN. The Lhx6^+^ projections are more likely to cluster around the rostral and ventrocaudal of STN (Mastro et al., 2014). In addition to PV^+^ and Lhx6^+^ neurons, very few Npas1^+^ neurons also send axons to STN (Hernández et al., 2015).

The GPe is a major afferent to GPi (or EPN) and different prototypic subpopulations have their own biased EPN subclass projections (Wallace et al., 2017). The ventral-anterior lateral thalamus-projecting EPN neurons receive the majority of their inputs from PV^+^ GPe neurons, while the lateral habenula-projecting EPN neurons receive more than half of their GPe inputs from PV-negative (PV^−^) neurons (Wallace et al., 2017).

Not every prototypic neuron gives rise to axons reaching all downstream nuclei. Some PV^+^ neurons selectively send axons to the STN and GPi, but not SNr (Mallet et al., 2012). The projections to SNr are diffusely distributed, without a clear topography (Mastro et al., 2014; Oh et al., 2017). The total amount and distribution of GPe terminals between PV^+^ and Lhx6^+^ neurons are similar, and both form basket-like synapses around the soma of SNr neurons (Mastro et al., 2014). Since SNr neuron subpopulations have different connectivity and functionality (Rizzi and Tan, 2019), whether there are preferential connections with distinct GPe neuron subtypes is worthy of further investigation. GPe neurons also form axons to the SNc, forming basket-like appositions with the soma of dopaminergic neurons (Oh et al., 2017). Compared to PV^+^ neurons, the Lhx6^+^ neurons project more heavily to the SNc (Oh et al., 2017). In contrast, the PV^+^ neurons preferentially project to the ventral tier of SNc, particularly along the border region between SNc and SNr (Mastro et al., 2014; Evans et al., 2020). There are multiple neuron subpopulations in the SNc (Poulin et al., 2018; Wu et al., 2019). Among them, aldehyde dehydrogenase 1A1-positive (ALDH1A1^+^) neurons are a group of DA neurons mainly located in the ventral tier of SNc that are preferentially affected in PD (Liu et al., 2014; Wu et al., 2019). However, there is still a lack of research directly examining connections between the GPe PV^+^ neurons and SNc ALDH1A1^+^ DA neurons. Delineation of the precise connections between these two distinct subpopulations of GPe and SNc neurons will facilitate a better understanding of their functional roles.

#### BG Upstream Nuclei

Arkypallidal neurons make up the bulk of the pallidostriatal projections to the striatum (Abdi et al., 2015; Hernández et al., 2015; Saunders et al., 2016). Unlike the prototypic neurons, arkypallidal neurons exclusively project to the dorsal striatum with thousands of axonal boutons (Mallet et al., 2012; Fujiyama et al., 2016) (Figure 3B). The Npas1^+^ neurons, presented as a mixture of arkypallidal and prototypic GPe neurons at 2:1 ratio, constitute the principle GPe inputs to SPNs and interneurons (Mallet et al., 2012; Hernández et al., 2015; Glajch et al., 2016; Saunders et al., 2016). The synaptic inputs from Npas1^+^ neurons are preferentially located on the dendritic regions of SPNs, and terminate closer to the soma in iSPNs than in dSPNs (Glajch et al., 2016). The PV^+^ neurons account for a minor fraction of the pallidocortical projections, a subset of which also project to the STN and SNr (Saunders et al., 2016). Such selective PV^+^ pallidostriatal axons potently inhibit interneurons, but rarely affect the SPNs (Mastro et al., 2014; Glajch et al., 2016; Saunders et al., 2016). This connectivity provides the structural basis for potential coordination between the STN and striatal interneurons. Compared with PV^+^ neurons, the Lhx6^+^ neurons project more densely to the dorsolateral striatum; however, the projections in striatum are generally sparse from both the PV^+^ and Lhx6^+^ neurons (Mastro et al., 2014).

#### Other Nuclei

Like the afferent projections, GPe neurons also give rise to projections outside of BG, including the cortex, reticular thalamic nuclei (RTN), Pf, lateral habenula, and amygdala (Saunders et al., 2015; Oh et al., 2017; Abecassis et al., 2020). Data indicate that the ChAT^+^ and some ChAT-negative (ChAT^−^) GPe neurons project to the frontal cortex (Mckinney et al., 1983; Mesulam et al., 1984; Chen et al., 2015; Saunders et al., 2015; Abecassis et al., 2020) (Figure 3B). Although the ChAT^+^ neurons belong to the cholinergic system, they still co-release GABA (Tkatch et al., 1998; Saunders et al., 2015). The ChAT^+^ neurons appear to arborize heavily in layer 1–3, while the ChAT^−^ neurons prefer to arborize in layer 5 and 6 (Saunders et al., 2015; Abecassis et al., 2020). One recent study has shown that ChAT^−^ neurons are likely to be a subset of neurons with both Npas1 and Nkx2.1 expression (Abecassis et al., 2020). It remains to be determined whether there are other GPe subtypes projecting to cortex. Besides the BG nuclei and cortex, Npas1^+^ and Nkx2.1^+^ GPe neurons also project to the RTN (Saunders et al., 2015; Abecassis et al., 2020). Since the RTN controls the overall information flow between the thalamus to cortex and activating GPe can diminish the firing rates of RTN neurons, it is possible that this group of GPe neurons gate the information between cortex and RTN (Villalobos et al., 2016).

### Local Circuitry in GPe

Regardless of GABAergic or cholinergic neurons, almost every GPe neuron generates local axon collaterals (Sadek et al., 2007; Mallet et al., 2012). Juxtacellular labeling indicates that local inhibitory synapses are located on the soma and proximal dendrites of GPe neurons (Sadek et al., 2007). The neurons in the inner GPe possess approximately twice as many axonal boutons (on average 581) as the neurons along the border with striatum (Sadek et al., 2007). In addition, the prototypic neurons generate longer local collaterals and a larger number of axonal boutons than the arkypallidal neurons (Mallet et al., 2012). The average number of local collateral boutons for each prototypic neuron is almost 500, while for each arkypallidal neuron there are only about 120 (Mallet et al., 2012; Fujiyama et al., 2016). These axon collaterals construct a complex network with local reciprocal GABAergic influence. Connections from prototypic to arkypallidal neurons are more abundant than connections from arkypallidal to prototypic, which allows prototypic axon collaterals function as a switch to control arkypallidal neuronal activity (Nevado-Holgado et al., 2014; Aristieta et al., 2020). On the other hand, local connections between arkypallidal neurons are modest, and the connections between prototypic neurons are even weaker (Nevado-Holgado et al., 2014). In PD, a hallmark of BG dysfunction is the amplification of synchrony, particularly in the form of β oscillation (a neural wave with a frequency range of 13–30 Hz) (Cruz et al., 2011; de la Crompe et al., 2020; Meng et al., 2020). It has been suggested that intrapallidal collaterals could participate in control of the firing rates, firing patterns and synchronization of GPe neurons (Miguelez et al., 2012), thus enabling the GPe to act as a central generator for β oscillation.

### GPe Networks in Normal State and Parkinson's Disease

**Figure 4:**
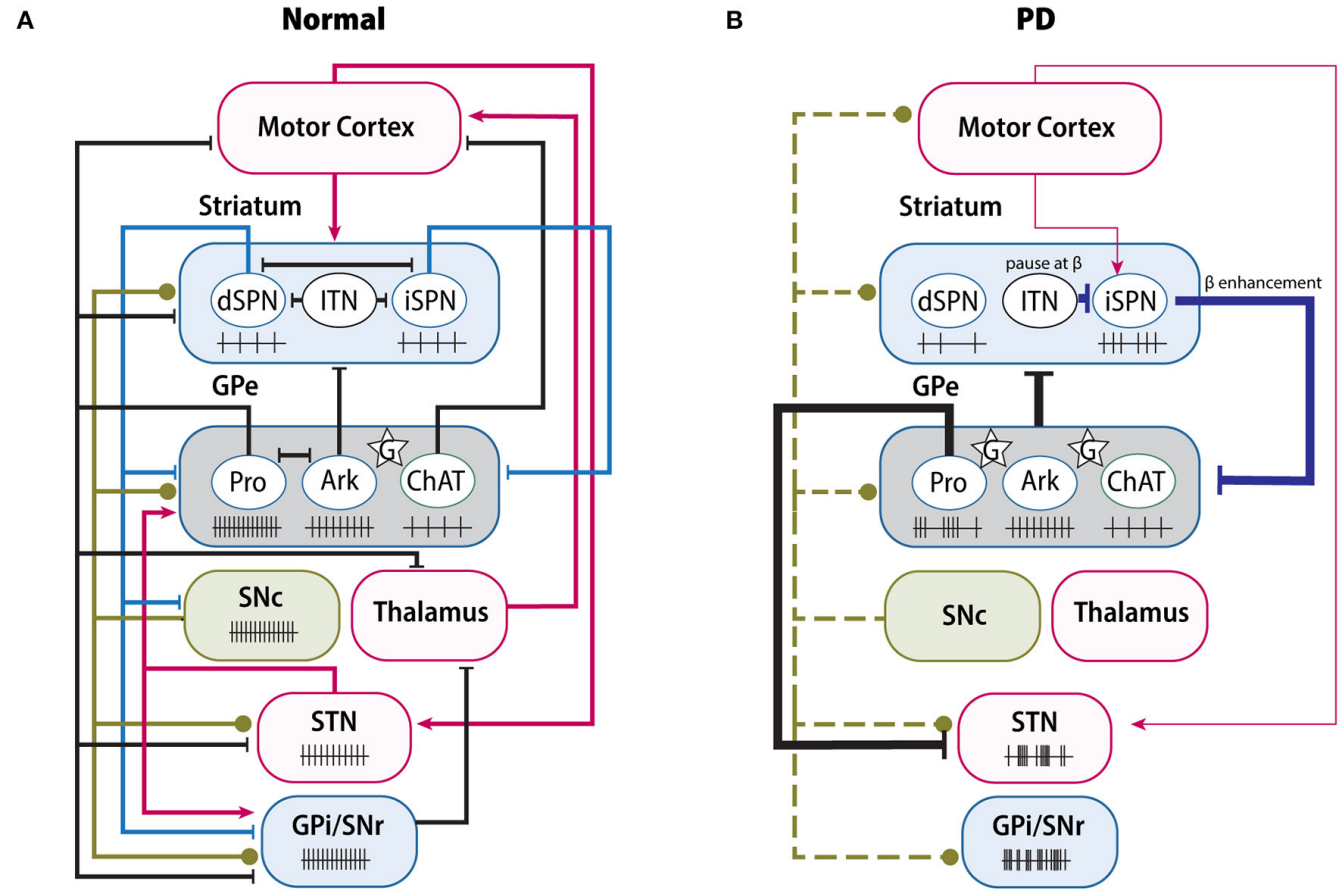
Changes of BG connectivity and neuron activity between normal and PD. **(A)** Complex functional connectivity and related neuron firing rate in normal condition. GPe receives the GABAergic projections from iSPNs and dSPNs (blue lines), the dopaminergic projections from SNc (green lines), and glutamatergic projections (red lines) from STN, cortex and thalamus. In normal condition, GPe neurons maintain their own firing rate; but in PD **(B)**, due to the loss of dopaminergic inputs (green dash lines), several pathways have been altered greatly, either enhanced or weakened, resulting in abnormal firing rate or pattern of both GPe and its downstream nuclei, such as increase of β oscillation. Ark, arkypallidal neurons; ChAT, choline acetyltransferase; G (star), glia; ITN, interneurons; Pro, prototypic neurons.

Based on the connectivity, the GPe networks can be divided into four parts: the GPe-striatum, GPe-STN, GPe-SN, and GPe-cortex networks. Given that both SNc and SNr belong to SN and there are connections between these two SN nuclei (Rice and Patel, 2015; Rizzi and Tan, 2019), we will discuss them together as the “GPe-SN network.” While we only describe four networks here, it is worth noting that the functional organization of the GPe and network relationships are more complex than just the simple reverberating feedback loops. We hope this section will provide a useful framework for appreciating GPe neuron activity and functions within the BG (Figure 4).

#### GPe-Striatum Network

In the BG circuits, the striatum mainly receives glutamatergic inputs from cortex and dopaminergic inputs from SNc, and sends GABAergic axons to GPe or downstream output nuclei (Calabresi et al., 2014). The direct and indirect pathways have been classically thought of as functionally opposing neural circuits engaged with selecting and refining motor performance (Grillner and Robertson, 2016). Optogenetic or chemogenetic stimulation of iSPNs can lead to massive inhibition of the activity of most GPe neurons and suppression of locomotion (Kravitz et al., 2010; Cazorla et al., 2014; Chu et al., 2015; Yuan et al., 2017; Bouabid and Zhou, 2018). However, a recent study from Aristieta et al. shows that activation of iSPNs can lead to a disynaptic excitation of arkypallidal neurons, which caused by the reduction of inhibition from prototypic axon collaterals (Aristieta et al., 2020). Stimulation of dSPNs can also inhibit the GPe neuron activity with the same latency as the activation of iSPNs (Cazorla et al., 2014). Increasing the excitability or expression of DRD2 could promote the growth of bridging collaterals from dSPNs into GPe (Cazorla et al., 2014). Interestingly, in DRD2-overexpressing mice, as the collaterals from dSPNs are increased, dSPNs stimulation can also result in decreased locomotor activity (Cazorla et al., 2014). Thus, the bridging collaterals in GPe have profound potential to regulate the coordination of the direct and indirect pathways.

The classical model predicts increased output of the indirect pathway in PD (Albin et al., 1989). Compelling evidence has revealed that the striatopallidal pathway is remodeled in a DA-depleted (DD) condition. In terms of morphology, the synaptic boutons of striatopallidal projections are 64% larger in the 6-hydroxydopamine (6-OHDA)-lesioned rat model than in naïve rats (Ingham et al., 1997). Due to the imbalance between the hyperactive iSPNs and hypoactive dSPNs following the loss of dopaminergic inputs (Ketzef et al., 2017; Parker et al., 2018), the increased iSPNs activity can suppress GPe (Kita and Kita, 2011; Mallet et al., 2012; Deffains et al., 2016). Furthermore, local astrocytes also participate in the regulation of GABAergic transmission of striatopallidal pathway (Cui et al., 2016; Chazalon et al., 2018). On the one hand, GPe astrocytes critically control the homeostatic glutamate content. In the DD condition, astrocytes reduce the ambient glutamine level, which in turn disinhibits the presynaptic GABA release from the striatopallidal synapse through mGluR3 (Cui et al., 2016). On the other hand, glial GABA transporter GAT-3 is downregulated by DD, leading to an elevation in extracellular GABA concentration, which induces the GABA_A_ receptor-mediated tonic inhibition on GPe neurons (Chazalon et al., 2018). More importantly, intrapallidal blockage of GAT-3 in normal mice can result in significant impairment of rotarod performance, suggesting that local glia dysfunction indeed contributes to PD pathophysiology and disease phenotypes (Chazalon et al., 2018). It should be noted that several compensatory homeostatic changes are also involved in regulating the indirect pathway, in order to balance the hyperactivity of iSPNs by DD. Increased inhibitory connectivity from interneurons and reduced afferent cortical glutamatergic synapses have been found selectively in iSPNs (Day et al., 2006; Gittis et al., 2011; Ztaou and Amalric, 2019). Taken together, the hyperactivity of the striatopallidal pathway is a consequence of multi-level modulation and is counterbalanced by several global homeostatic alterations.

Under normal conditions, GPe neurons have their own firing rates and firing pattern, but in the DD condition, these firing patterns are interrupted (Deister et al., 2013; Abdi et al., 2015). Prototypic neurons are the most affected population in anesthetized rats, presenting phase-locked activity and pauses (Abdi et al., 2015; Kovaleski et al., 2020). Several studies have claimed that the pauses are attributable to burst activation of iSPNs (Kita and Kita, 2011; Sharott et al., 2017), because optogenetic inhibition of iSPNs can ameliorate the abnormal pause of PV^+^ neurons in GPe (Kovaleski et al., 2020). However, the firing rate of prototypic neurons in DD is not consistent over different studies, presenting either no change or being significantly decreased (Kita and Kita, 2011; Hernández et al., 2015; Glajch et al., 2016), which may be attributed to the compensatory homeostatic synaptic or cellular inputs from other nuclei, such as the STN (Wichmann and DeLong, 2006; Kita and Kita, 2011). In contrast, arkypallidal neuron firing is not obviously impacted by SPNs in the DD condition. They continue to fire in time, only showing an increased phase-coupling pattern (Abdi et al., 2015). Overall, the disparate connectivity between distinct groups from the striatum and STN may contribute to the difference between prototypic and arkypallidal neuron activity in the DD condition (Nevado-Holgado et al., 2014). Reciprocally, GPe neurons provide inhibitory inputs to both GPe interneurons and to SPNs in the dorsal striatum (Abdi et al., 2015; Hernández et al., 2015; Saunders et al., 2016). In the pallidostriatal pathway, arkypallidal neurons exert a suppressive role in motor initiation (Mallet et al., 2016). Glajch et al. indicated that after stimulating the terminal field of the pallidostriatal axons, both iSPNs and dSPNs exhibit decreased firing, especially in chronic 6-OHDA-lesioned mice (Glajch et al., 2016). Under the DD condition, although the inhibitory postsynaptic current amplitudes are increased in both SPN classes, the paired pulse ratio is unchanged (Corbit et al., 2016; Glajch et al., 2016), suggesting there exists postsynaptic regulation of GABA transmission in SPNs. It is noteworthy that in addition to targeting SPNs, the GPe also projects to striatal interneurons (Mallet et al., 2012; Mastro et al., 2014; Saunders et al., 2016). Several studies even argue that the functional connections from GPe onto fast-spiking interneurons (FSIs) are substantially stronger than onto SPNs (Bevan et al., 1998; Corbit et al., 2016). Striatal interneurons have been considered the main contributor to synchronization and focused rhythmicity within striatum (Berke et al., 2004; Gittis et al., 2011; Kondabolu et al., 2016). By using electrophysiological tests and computational analysis, Corbit et al. proposed a mechanism for the enhanced β oscillation based on the GPe-FSIs-SPNs loop (Corbit et al., 2016). They claimed that in the DD condition, the synchronized GPe neurons can pause FSIs to β frequencies and entrain SPNs firing to β band rhythms, which in turn enhances synchronized GPe spikes as a β oscillation through the striatopallidal pathway. Therefore, this may represent a positive feedback loop between the GPe and striatum via striatal interneurons.

#### GPe-STN Network

Topographically, GABAergic GPe neurons and glutamatergic STN neurons form a reciprocal loop in the BG. The STN also receives monosynaptic glutamatergic inputs from the cortex (Nambu et al., 2002), as an intersection between indirect and hyper-direct pathways. Under normal conditions, the hyper-direct pathway balances the synaptic excitation and inhibition of STN by regulating the strength of GABAergic pallidosubthalamic transmission (Chu et al., 2015, 2017). As discussed above, the hyperactivity of iSPNs leads to greater inhibition of GPe neurons, and this is supposed to disinhibit the STN in PD. However, recent studies argue that GABAergic transmission from GPe to STN is greatly augmented in the DD condition. On one hand, the number of synaptic connections per pallidosubthalamic axon terminal is significantly increased following DD (Fan et al., 2012; Chu et al., 2015). Chu et al. reported that the abnormal strengthening of the GPe-STN connection leads to the disinhibition of STN and excessive activation of STN NMDA receptors (Chu et al., 2015). In parallel, cortical synaptic transmission to STN is also reduced, which induces the maladaptive reduction of STN autonomous firing (Mathai et al., 2015; Wang et al., 2018; McIver et al., 2019). Knockdown of STN NMDA receptors can significantly suppress the GABAergic pallidosubthalamic proliferation and improve motor functions by DD (Chu et al., 2015, 2017). On the other hand, the pallidosubthalamic pathway is also regulated by presynaptic DRD2-class receptors (Baufreton and Bevan, 2008). Theoretically, loss of DA may make GABAergic pallidosubthalamic inputs more powerful in regulating the STN neuron activity, but this hypothesis still needs to be confirmed in DD models. In general, the frequency and pattern of STN activity remain unchanged or even suppressed in DD mice (McIver et al., 2019; Kovaleski et al., 2020), and are not hyperactive (Breit et al., 2006; Mehler-Wex et al., 2006). Homeostatic synaptic and cellular plasticity may participate in stabilizing STN neuron activity in PD (Chu et al., 2015, 2017; Wang et al., 2018; McIver et al., 2019).

The STN provides glutamatergic transmission to both prototypic and arkypallidal neurons. Optogenetic inhibition of STN neuron activity or intrapallidal application of AMPA and NMDA receptor blockers can globally reduce GPe activity (Kita et al., 2004; Kovaleski et al., 2020), but specific effects may be different between the prototypic and arkypallidal neurons. Two studies claimed that optogenetic stimulation or inhibition of the STN preferentially stimulates or inhibits the prototypic neurons, but has an opposite impact on the arkypallidal neurons (Aristieta et al., 2020; de la Crompe et al., 2020). The disynaptic inhibition of arkypallidal neurons are likely from the innervation of prototypic axon collaterals (Mallet et al., 2012; Aristieta et al., 2020). Under the DD condition, both AMPA and NMDA receptors are found downregulated in the GPe (Porter et al., 1994; Betarbet et al., 2000) and the ambient glutamate content is decreased following chronic DA loss (Cui et al., 2016). Recent data also show that the STN inputs to PV^+^ neurons are reduced in 6-OHDA-lesioned mice (Pamukcu et al., 2020). These alterations are relatively consistent with the low STN neuron activity in PD (McIver et al., 2019). Additionally, the STN is an important target of deep brain stimulation (DBS) for mid- or late-stage PD patients (Odekerken et al., 2016). However, the mechanism behind DBS remains to be clarified. Preclinical and clinical data confirm that high frequency electrical stimulation of STN can improve motor symptoms of PD, but it is still unknown if it results from an increase or decrease of STN activity (Chiken and Nambu, 2016). Several studies show that STN high frequency stimulation can increase the pallidal ambient glutamate level and average firing rates of GPe neurons (Windels et al., 2000; Reese et al., 2011). In general, the cell-specific effects of both the pallidosubthalamic and reciprocal pathway are still not clear and need to be further investigated.

The GPe-STN network is a key player in β oscillation (Plenz and Kital, 1999; Koelman and Lowery, 2019; de la Crompe et al., 2020). Both GPe and STN neurons are able to fire action potentials in an autonomous manner, which makes their spiking activity independent of excitatory inputs (Surmeier et al., 2005; Mallet et al., 2019). Under normal conditions, the GPe-STN network exhibits decorrelated and irregular activity (Magill et al., 2000; Elias et al., 2007; Mallet et al., 2008a), whereas in idiopathic PD and experimental models, GPe and STN are strongly correlated and produce pausing and bursting activity (Walters et al., 2007; Mallet et al., 2008b; Delaville et al., 2015). Usually, β oscillation is not exaggerated until several days after 6-OHDA lesion (Mallet et al., 2008b), suggesting it needs a long-term adaptive change of the synaptic alteration. There are many hypotheses concerning the generation and propagation of abnormal β oscillation activity in PD (Walters et al., 2007; Shouno et al., 2017; Bahuguna et al., 2020). Several studies have suggested that the intrinsic β oscillation in the GPe-STN network is too weak to act as a pacemaker and the β oscillation might be generated by cortex, and later amplified by the GPe-STN network (Brittain and Brown, 2014; Koelman and Lowery, 2019). However, a more recent study indicates that this abnormal neural dynamic is critically dependent on GPe rather than on cortex and STN (de la Crompe et al., 2020). In line with the above hypothesis, another new study has shown that the bursting of STN depends on the bursting of GPe neurons (Bahuguna et al., 2020). Taken together, we believe that the GPe-STN network is important in the maintenance and propagation of β synchronization.

#### GPe-SN Network

The GPe receives dopaminergic inputs from the SNc and in turn sends inhibitory GABAergic efferents to both the SNc and SNr. Although the nigral inputs to the GPe may seem negligible compared with nigrostriatal inputs, they still exert a profound effect in regulating the GPe activity and motor functions. Several studies have demonstrated that intrapallidal infusion of DA can increase the firing rate of a majority of GPe neurons (Querejeta et al., 2001; Mamad et al., 2015). This increased firing rate can be mimicked by infusing DRD2 agonist and prevented by DRD2 antagonist (Querejeta et al., 2001). Moreover, local DA depletion or DRD2 blockage also influence the firing rate or firing pattern of the downstream nuclei and lead to motor deficits (Querejeta et al., 2001; Bouali-Benazzouz et al., 2009; Abedi et al., 2013; Mamad et al., 2015). In PD, due to the loss of dopaminergic neurons in the SNc, GPe DA level has been found to be significantly decreased in both PD patients and experimental models (Parent et al., 1990; Rajput et al., 2008). Pallidal infusion of DA can partially restore the motor deficits in 6-OHDA-lesioned rats (Galvan et al., 2001). Several recent radiological studies on PD patients also confirmed alterations of the nigropallidal pathway, reflected in the reduction of vesicular monoamine transporter 2 (VMAT2) expression in the GPe (Cho et al., 2019) and connectivity changes were seen using deterministic tractography (Tan et al., 2015). However, since DA receptors also exist on the striatopallidal axons terminals (Smith and Villalba, 2008), simple intrapallidal DA infusion or examination cannot precisely differentiate the exact action sites in the GPe. Theoretically, activating DRD2 on the striatopallidal axons can also reduce GABAergic release, which will lead to the disinhibition of GPe neurons as well. As introduced before, DRD2 is widely expressed in GPe neurons and more concentrated in the striatum-projecting neurons (Hoover and Marshall, 2004), which means there may exist a bias of dopaminergic modulation on arkypallidal neurons more than prototypic neurons. Therefore, further investigations of cell-specific dopaminergic effects on GPe neurons are warranted.

Anatomically, the GPe also directly innervates both the SNc and SNr (Mastro et al., 2014). These nuclei are predominantly or exclusively regulated by GABA_A_ receptors (Brazhnik et al., 2008; Evans et al., 2020). The inhibitory effect on SNc neurons between GPe subpopulations are different. When compared with PV^+^ neurons, the Lhx6^+^ neurons can lead to a stronger inhibition of tonic firing and hyperpolarization of SNc neurons (Evans et al., 2020). However, the exact physiological functions of the pallidonigral pathway remain to be determined. For example, questions of whether the GPe activity is related to DA release dynamics or what factors influence the pallidonigral pathway remain to be clarified in the future. As an output nucleus in the BG, the SNr receives GABAergic inputs from both the striatum and GPe (Evans et al., 2020; Phillips et al., 2020). The pallidal projections engage in tonic spiking activity and paired-pulse depression on SNr neurons (Connelly et al., 2010). It has been reported that stimulating DRD4 on the pallidal projections can inhibit the GABA release on the SNr and consequently reduce motor activity (Erlij et al., 2012). Computational analysis predicts that in the DD condition, the depressing GPe-SNr synapses make SNr neurons become more sensitive to irregular GPe firing (Lindahl et al., 2013). Variance of GPe activity could modulate the low-frequency oscillations and synchrony to spread through SNr neurons (Phillips et al., 2020). In PD, a hallmark abnormality of SNr neurons is the increase of burst firing (Rubin et al., 2012). Recent studies have indicated that distinct GPe subpopulations can have contrasting effects on SNr neuron activity and behavioral functions (Mastro et al., 2017; Pamukcu et al., 2020). By using intrapallidal optogenetic stimulation, Mastro et al. demonstrated that either activation of PV^+^ neurons or inhibition of Lhx6^+^ neurons could induce a persistent reduction in the burst firing of the SNr and restore movement in DD mice (Mastro et al., 2017). Additionally, activating PV^+^ neurons leads to an inhibitory effect on PV^−^ neurons, forming a dissociating effect on different subpopulations in the GPe (Mastro et al., 2017). Given that the SNr may receive pallidal instructions indirectly via STN, Pamukcu et al. chose to specifically stimulate PV^+^ axon terminals in the SNr and found that this intervention also enhances movement velocity in naïve mice (Pamukcu et al., 2020). Together, these data suggest that the GPe produces a cell-specific effect on SNr neurons. Interestingly, it has been proposed that GABAergic synaptic transmission in SNr may not only be purely inhibitory, but may be effectively excitatory in some neurons (Phillips et al., 2020). In addition, GPe local axon collaterals have the capability to inhibit neighboring neurons, which could partly explain the dissociating phenomenon in GPe neurons. In addition, it has been shown that distinct SNr subpopulations control different domains of motor behavior (Rizzi and Tan, 2019). Whether different GPe subpopulations have their own inclination toward distinct targets of SNr neurons and result in distinct motor effects remain to be determined.

#### GPe-Cortex Network

Classical models postulate that the BG modulates the cortex by acting on thalamus through direct and indirect pathways, and cortex commands the BG through the input structure of the striatum (Smith et al., 1998). In fact, both anatomical and electrophysiological examinations have confirmed that there exists a direct loop between the cortex and GPe (Saunders et al., 2015; Karube et al., 2019; Abecassis et al., 2020). The cortex provides monosynaptic glutamatergic inputs to the GPe, particularly to the striatum-projecting neurons (Karube et al., 2019; Abecassis et al., 2020). Comparing cortical excitatory postsynaptic currents arriving at STN and at GPe neurons, both have a similar weights, but the rise time and decay constant in excitatory postsynaptic currents at GPe neurons tend to be longer (Karube et al., 2019). As for β oscillation formation, because cortex is a potent rhythm generator and presents abnormal oscillation in PD (Steriade, 2006; Pollok et al., 2012), it is possible that the direct corticopallidal connection could promote the formation and propagation of β oscillations in the GPe. Reciprocally, optogenetic experiments have shown that the GPe can rapidly and potently sculpt the firing activity of frontal cortex neurons (Saunders et al., 2015; Guo et al., 2016). Among the cortex-projecting GPe neurons, ChAT^−^ neurons only send inhibitory GABAergic projections to cortical interneurons or pyramidal neurons, whereas ChAT^+^ neurons are able to produce both inhibitory and excitatory effects on the cortex via releasing GABA and acetylcholine (Saunders et al., 2015). However, the mechanism underlying this dual transmitter release of ChAT^+^ neurons has yet to be fully developed. Because GPe neurons also receive innervations from the STN and striatum, the GPe-Cortex network may allow direct, indirect and hyper-direct pathways together to modulate the cortex in concert (Saunders et al., 2015). It will be of great interest to further examine the properties of this network, especially as it relates to the synaptic transmission, neuron activity and behavioral alterations in PD.

### The Role of GPe in Parkinson's Disease

PD is clinically characterized by a range of motor and non-motor symptoms (Sveinbjornsdottir, 2016). Many PD symptoms can be directly attributed to dopaminergic neurodegeneration, while others appear to be driven by altered connectivity or aberrant neuron activity of BG nuclei (McGregor and Nelson, 2019). GPe is centrally positioned in the BG and can directly or indirectly connect to almost all the nuclei related to PD relevant behaviors. It also participates in regulating the normal activity or abnormal synchronization and oscillation of the BG. Therefore, we focus on the GPe-related underlying mechanisms of PD motor and non-motor symptoms and discuss the potential GPe-based treatments for PD.

#### GPe-Dependent Mechanisms of PD Symptoms

The cardinal motor symptoms of PD are bradykinesia, tremor, rigidity and postural instability (Sveinbjornsdottir, 2016). As for the classical models, following the loss of DA, imbalance in direct and indirect pathways leads to the inhibition of the GPe and disinhibition of the BG output nuclei, which further suppress the thalamus and cortex, and hence contribute to the akinesia, bradykinesia and rigidity in PD (Albin et al., 1989; Surmeier et al., 2014; Nambu et al., 2015; Moustafa et al., 2016; Mallet et al., 2019; McGregor and Nelson, 2019). Since GPe is the direct downstream target of iSPNs and dSPN collaterals, its dysfunction is believed to be involved in almost all the cardinal symptoms of PD. Researchers use locomotor tests to examine velocity and mobile or immobile durations in PD rodent models, as these features recapitulate the bradykinesia and akinesia symptoms of PD patients. Most studies have shown locomotion changes after genetic or pharmaceutical intervention in the GPe as discussed before (Abedi et al., 2013; Mastro et al., 2017). Additionally, one earlier study demonstrated that blockage of excitatory innervations in the GPe induces rigidity in rats by examining their catalepsy time (Turski et al., 1990). Besides bradykinesia and rigidity, tremor is also common in PD patients, usually occurring during rest with a rhythm between 4 and 6 Hz (Sveinbjornsdottir, 2016). Resting tremor is thought to be caused by pathological alterations in the connectivity between the BG and cerebellothalamic circuits (Helmich et al., 2011). It has been found that DA depletion in the GPe correlates to the severity of tremor in PD patients (Helmich et al., 2011). Given that the Pf is related to tremor controlling and there exists a reciprocal connection between the GPe and Pf, it is possible that the GPe dysfunction can induce tremor through the Pf in the thalamus (Krauss et al., 2002). On the other hand, neuron recording in PD patients reveals there is oscillatory synchronization of the GPe and GPi appearing in patients with limb tremor (Levy et al., 2002).

A commonly studied pathology in PD is the abnormal firing pattern and excessive β oscillation in the BG nuclei. Due to the technical differences in research approach or to compensatory mechanisms, the firing rate alteration can be moderate or even opposite to what classical models predict in PD (Mallet et al., 2019). Thus, we cannot simply use classical models to fully explain mechanisms underlying PD symptoms. In terms of the firing pattern, burst firing has been observed in GPe neurons in both PD patients and experimental models. This abnormal burst firing can be reduced by using DA replacement therapy, and its reduction parallels the improvement of motor deficits (Filion and Paul, 1991; Kita and Kita, 2011; Singh et al., 2015). In addition, medication and DBS can reduce the excessive β oscillation in PD patients (Cruz et al., 2011; Kumar et al., 2011; de la Crompe et al., 2020; Meng et al., 2020). This increased β oscillation has been linked with bradykinesia, akinesia and rigidity (Moustafa et al., 2016). The GPe-Striatum, GPe-STN, GPe-Cortex and GPe-SN, are each involved in the generation or propagation of β oscillation throughout the BG. There is no doubt that the GPe plays a prominent role in abnormal β oscillation expression. Mallet et al. have proposed that the intrapallidal GABAergic control is mainly achieved by GPe neurons under normal conditions. However, hyperactive iSPNs may operate a switch from intrinsic GABAergic control to extrinsic control, which leads to maladaptation of GPe neurons and generates the excessive β oscillation (Mallet et al., 2019; de la Crompe et al., 2020). Additionally, if we compared the number of boutons from the striatal and subthalamic afferents to GPe, it is not hard to conclude that striatum provides a more robust innervation to GPe neurons (Oorschot, 1996; Koshimizu et al., 2013; Kita and Jaeger, 2016), not to mention that the striatopallidal pathway becomes hyperactive in the DD condition. Moreover, the GPe-FSIs-SPNs loop could amplify the β oscillation in BG (Corbit et al., 2016). Therefore, it is more likely that the excessive β oscillation in the GPe is driven by the striatum, rather than STN. The GPe-STN network may play a role in the maintenance and propagation of β oscillation. However, whether the excessive β oscillation causes the PD symptoms or is just a phenomenon accompanying the symptoms is still a question worth to be further clarified (McGregor and Nelson, 2019).

In addition to the cardinal symptoms, PD patients often have difficulties in self-initiating and executing movements (Wu et al., 2011, 2015). Flexible behavior requires the coordination of action initiation and suppression, also known as the “Go” and “Stop” cues (Bari and Robbins, 2013). There are several pathways involved in “Stop” cue processing. The STN responds very quickly to the “Stop” cue, which is enough to inhibit action (Schmidt et al., 2013). The GPe is also implicated in action suppression and stopping (Aron, 2011; Freeze et al., 2013; Sano et al., 2013), encoding the no-reward outcome and next-action selection (Nonomura et al., 2018). The arkypallidal neurons can also send a “Stop” sign and suppress current inappropriate movements through the pallidostriatal pathway (Mallet et al., 2016; Yoshida and Tanaka, 2016). Recently, Gu *et al*. confirmed that the GPe mediates proactive inhibition during the preparation stage of stop (Gu et al., 2020). Taken together, it is possible that GPe dysfunction could lead to the degradation of a neural “Stop” signal, and consequently to the movement initiation or canceling problems in PD.

Non-motor symptoms normally present much earlier onset than the typical motor symptoms in PD (Poewe, 2008). Current studies indicate that GPe dysfunction is correlated with non-motor symptoms including sleep disorder, anxiety and depression (Yuan et al., 2017; Avila et al., 2020). Sleep disorder manifests as deficiencies in slow-wave sleep, in both rapid eye movement and non-rapid eye movement stages, and affects up to 80% of PD patients (Friedman and Millman, 2008; Castillo et al., 2020). Multiple experiments have indicated that the BG plays a role in sleep-awake regulation (Qiu et al., 2010; Rolinski et al., 2016). GPe lesions cause a profound loss of sleep, and optogenetic or chemogenetic stimulation of the striatopallidal pathway can promote sleep duration, specifically in the non-rapid eye movement stage (Qiu M.-H. et al., 2016; Yuan et al., 2017). Interestingly, lesions in the STN only minimally effect the sleep-wake cycle (Qiu et al., 2010), indicating that while the GPe directly conjugates iSPNs in sleep-wake regulation, other nuclei may also influence sleep behavior. Another common non-motor symptom of PD is anxiety, which is reported to affect ~40% of PD patients (Pontone et al., 2009). Avila et al. indicated that the pallidal 6-OHDA-lesioned rats exhibit not only motor deficits, but also anxiety and depression disorders, and that anxiety can be improved by local administration of dopaminergic agents in the GPe (Avila et al., 2020). In conclusion, GPe dysfunction is associated with almost all the motor and non-motor symptoms in PD. Understanding the cell-type and circuit mechanisms underlying these symptoms is the foundation for better diagnosis and treatment of PD.

#### GPe-Dependent Applications for PD Diagnosis and Treatment

With the technological advances in the field of radiology, the molecule- or network-level changes in the brain can be visualized with a wide range of diverse techniques. For example, by using positron emission tomography, some molecules, like VMAT2, are found reduced in the GPe regardless of the disease stage (Cho et al., 2019). Additionally, functionally positive or negative correlation between the different GPe networks have been detected through functional magnetic resonance imaging (Rodriguez-Sabate et al., 2019). Therefore, it is possible to link the GPe network state with PD diagnosis. Given that early diagnosis is critically important for PD (Le et al., 2017), further investigations on GPe dysfunction in prodromal or even preclinical phases will be needed to significantly improve PD diagnosis.

As for PD treatment, DBS has been widely accepted as an effective neurosurgical method for PD. The STN and GPi are the most commonly used targets to treat motor symptoms in advanced PD patients (Chiken and Nambu, 2016; Dong et al., 2016). The GPe is also a potential target for DBS and has been tested in both animal models and PD patients. During GPe-DBS, bradykinesia has been found to significantly improve in both PD patients and non-human primates (Vitek et al., 2004, 2012). The effects of GPe-DBS are likely due to the firing pattern changing in the GPi and STN through stimulating striatopallidal axons or GPe neurons (Vitek et al., 2012). The safety and feasibility of GPe-DBS has been supported by a recent preliminary experiment from the Mayo Clinic (Castillo et al., 2020). More importantly, they have found that GPe-DBS improves sleep quality, which cannot be treated by GPi- or STN-DBS in PD patients (Castillo et al., 2020). This effect is consistent with the results from animal experiments (Qiu M. et al., 2016; Qiu M.-H. et al., 2016; Yuan et al., 2017). There are still several concerns about the application of GPe-DBS. First, due to the heterogeneity of GPe neurons, GPe-DBS produces complex responses composed of both excitation and inhibition of GPe neurons (Chiken and Nambu, 2013; Mastro et al., 2017). How to shift the balance of specific GPe subpopulations to rescue motor function is an important topic for further exploration. Second, a few studies claim that GPe-DBS seems to more readily induce dyskinesias in PD patients than GPi-DBS (Vitek et al., 2004; Elkouzi et al., 2019). Together, the GPe is a promising DBS target for treating both motor and non-motor symptoms in PD, but more clinical trials are needed to further validate the application in clinic.

## Conclusions

With the revelation of the anatomical, molecular and electrophysiological diversity of GPe cell types, a clearer picture of GPe composition starts to emerge. Due to the high heterogeneity of GPe neurons, GPe emerges as a complex hub among other nuclei, not just a relay station for the indirect pathway. Insights into the GPe cell and circuit-level organization can help to better understand neuron activity and modulation within GPe. Despite the extensive literature discussed here, our understanding of how GPe neurons integrate into the whole-brain computation to control motor and non-motor behavior in both healthy and PD conditions is still limited.

Several major topics must be addressed as the field uncovers more about the role of GPe. First, there are still discrepancies concerning the expression and relationship among some molecular markers in the GPe, especially the overlapping Lhx6^+^ and PV^+^ subpopulations. How to normalize animal experiments, including operation protocols, animal gender, and age, is worthwhile to explore in the future. Second, the majority of previous studies about GPe are based on the assumption that the GPe is a homogenous structure. It will be important to continue to explore the connectivity and functionality of distinct GPe subtypes, like prototypic and arkypallidal neurons, in both normal and PD conditions by using genetic tools or more advanced techniques. Third, some molecules, such as the cannabinoid-1 receptors, have been found to be enriched in the GPe and to exert a critical role in PD development (Davis et al., 2018). How these molecules effect GPe cells in PD will be an interesting subject for further interrogation. Fourth, because it is hard to detect β oscillation in PD rodent models, whether β oscillation is necessary in the development of PD is worth evaluating using experimental animal models derived from multiple alternative species. Fifth, GPe is involved in the both motor and non-motor functions in PD, whether these two functions share a common pathological pathway in PD is worthy of investigation. Addressing these questions will be important to help us further understand how the GPe and whole BG works in the normal and disease conditions.

## Data Availability Statement

The original contributions presented in the study are included in the article/supplementary material, further inquiries can be directed to the corresponding author/s.

## Author Contributions

JD reviewed the literature and wrote the manuscript. SH, JW, and WL provided many useful suggestions and helped to edit this manuscript. HC conceived the idea of this project and was involved in the revising manuscript and acquisition of funding. All authors read and approved the final manuscript.

## Conflict of Interest

The authors declare that the research was conducted in the absence of any commercial or financial relationships that could be construed as a potential conflict of interest.

## Abbreviations

ALDH1A1^+^: Aldehyde dehydrogenase 1A1 positive
AMPA: α-amino-3-hydroxy-5-methylisoxazole-4-propionic acid
BG: Basal ganglia
ChAT: Choline acetyltransferase
DA: Dopamine
DBS: Deep brain stimulation
DD: DA-depleted
DRD1: D1 dopamine receptors
DRD2: D2 dopamine receptors
DRN: Dorsal raphe nucleus
dSPNs: Direct pathway SPNs
EPN: Entopeduncular nucleus
FoxP2: Forkhead box protein P2
FSIs: Fast-spiking interneurons
GATs: GABA transporters
GPe: Globus pallidus externa
GPi: Globus pallidus interna
iSPNs: Indirect pathway SPNs
Lhx6: LIM homeobox 6
mGluRs: Metabotropic glutamate receptors
Nkx2.1: Nk2 homeobox 1
NMDA: N-methyl-D aspartic acid
Npas1: Neuronal PAS domain protein 1
PD: Parkinson's disease
Pf: Parafascicular
PPN: Pedunculopontine tegmental nucleus
PV: Parvalbumin
RTN: Reticular thalamic nuclei
SNc: Substantia nigra pars compacta
SNr: Substantia nigra pars reticulata
SPNs: Spiny projection neurons
STN: Subthalamic nucleus
VMAT2: Vesicular monoamine transporter 2
VTA: Ventral tegmental area.
